# *Macleaya* Extract versus Pregabalin Therapeutic Effectiveness for Neuropathic Pain in Dogs

**DOI:** 10.1155/2024/9939754

**Published:** 2024-07-30

**Authors:** Saowanee Petchnamthong, Titiyaporn Krambunlue, Nirut Suwanna

**Affiliations:** ^1^ Department of Companion Animal Clinical Sciences Faculty of Veterinary Medicine Kasetsart University, Bangkok 10900, Thailand; ^2^ Med Daylight Co., Ltd, Bangkok 10240, Thailand

## Abstract

Dogs suffer from neuropathic pain due to several neurological disorders, particularly intervertebral disc disease. We aimed to identify alternative therapeutics for neuropathic pain relief in dogs by comparing the treatment efficacy of pregabalin and *Macleaya* extract against neuropathic pain from cervical spinal hyperesthesia caused by intervertebral disc disease in dogs. We evaluated 10 dogs suffering from cervical intervertebral disc disease for neuropathic pain using the filament von Frey aesthesiometer (VFA). The dogs were randomly divided into two groups, G1 (*n* = 5) was treated with 4 mg/kg of pregabalin/12 hours orally for 14 days, and G2 (*n* = 5) was treated with 15 mg/kg *Macleaya* extract once daily orally for 14 days. To detect neuropathic pain, the VFA was used to measure the sensory threshold (ST) on days 0, 7, and 14. The results revealed decreases in all ST outcomes with no significant differences between the groups on day 14 posttreatment, as well as a decrease in the severity of neurological disease in both groups on day 7 posttreatment. No significant differences were detected in hematological and biochemical profiles (alanine aminotransferase (ALT), alkaline phosphatase (ALP), serum creatinine (SCR), urea nitrogen (BUN), and total protein (TP)) in both groups between pre- and posttreatment were not significantly different. The *Macleaya* extract can reduce neuropathic pain in a similar pattern to pregabalin. *Macleaya* extract may be effective for the treatment of neuropathic pain in dogs with spinal hyperesthesia.

## 1. Introduction

Neuropathic pain is described as hypersensitivity to afferent and efferent stimuli (hyperalgesia and allodynia) caused by diseases or lesions of the somatosensory system [1]. The prevalence of neuropathic pain ranges between 3 and 17% in humans and 7-8% in dogs and cats [2]. Neuropathic pain can decrease sensory threshold (ST) and alter the function of sensory nerve fibers [3], which can lead to hyperesthesia.

Hyperesthesia refers to hypersensitivity of any sensory response, particularly pain, and the most common types of hyperesthesia are allodynia and hyperalgesia. Pregabalin, an antagonist of the *α*2*δ*-subunit (type I) of the calcium channel, has shown effectiveness in relieving neuropathic pain. This mechanism can decrease the calcium influx into the sensory neurons leading to an analgesic effect [4]. In dogs, pregabalin has been used to reduce neuropathic pain following spinal cord decompression surgery [5]. However, the use of pregabalin should be avoided in the presence of renal impairment [6].

*Macleaya cordata* is a traditional Chinese herb belonging to the genus *Macleaya* of the *Papaveraceae* family [7]. The main pharmacological component is protopine, which exerts its analgesic activity mainly through the activation of opioid receptors, inhibition of calcium ions, and the activation of alpha receptors [8]. *Macleaya* extract has been developed and used in veterinary medicine due to its wide therapeutic properties [9]. Other studies have shown that *Macleaya* extract can reduce inflammation [10] and inhibit nuclear factor-*κ*B (NF-*κ*B) activation [11]. An experiment in mice showed that inhibition of NF-*κ*B reduced the pain and inflammatory processes associated with nerve-end injury [12]. In addition, NF-*κ*B was found to affect substance P expression and nociceptive pain in the case of herniated discs [13], while inhibiting substance P can reduce neuropathic pain [14]. Thus, we evaluated the efficacy of ST measurements of neuropathic pain in responding to *Macleaya* extract and pregabalin treatment for spinal hyperesthesia in dogs. The safety consideration of these drugs was also demonstrated by hematological and blood chemistry tests.

## 2. Materials and Methods

### 2.1. Ethical Approval and Informed Consent

The research protocol was ethically approved by the Institutional Animal Care and Use Committee of the Kasetsart University, Bangkok, Thailand (protocol ACKU64-VET-060). Consent was obtained from all dog owners, and all procedures in this clinical trial complied with the Kasetsart University Institutional Animal Care and Use Standards.

### 2.2. Animals

**Figure 1:**
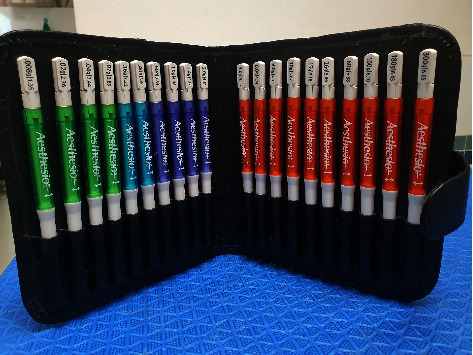
A set of filaments in von Frey aesthesiometer (Aesthesio®) was used for evaluating neuropathic pain.

**Figure 2:**
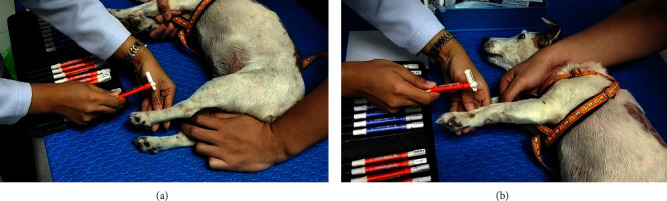
Application of von Frey aesthesiometer monofilament to evaluate the sensory threshold of limbs: application of the monofilament (a) on the dorsal surface of the left hindlimb between the fourth and fifth metatarsus; (b) with pressure until it was bent.

The study involved 10 male or female dogs of less than 30 kg of any breed and age visiting the neurology center at the Kasetsart University Veterinary Teaching Hospital at Bangkhen Campus, Faculty of Veterinary Medicine, Kasetsart University, Thailand. Dogs were partitioned using a random, double-blind procedure and divided into a pregabalin-treated group (G1, 4 mg/kg of pregabalin twice daily) and a *Macleaya*-treated group (G2, 15 mg/kg once daily). The dogs were excluded if they had a history of orthopedic disease on the preliminary examination, and the severity of paresis was more than grade 3. A filament von Frey aesthesiometer (VFA) was used to evaluate the ST for neuropathic pain [15]. The study used a set of von Frey precision tactile sensory evaluators (Aesthesio®, DanMic Global, LLC, USA) 20 monofilaments (Figure 1) covering a range of 0.008–300 g (0.008, 0.02, 0.04, 0.07, 0.16, 0.4, 0.6, 1, 1.4, 2, 4, 6, 8, 10, 15, 26, 60, 100, 180, and 300 g). The dogs were placed in a quiet examination room for five minutes. Then, the monofilament was pressed to the dorsal surface between the fourth and fifth metacarpus and metatarsus (Figure 2(a)) [3]. More pressure was gradually applied to the monofilament until the monofilament was bent (Figure 2(b)). The ST result was recorded when the plastic fiber was bent, and the animal produced a behavioral response, such as vocalization, leg pulling, licking, and trembling [16]. The severity of neurological sign evaluation was based on a modified Frankel score method to classify into 6 grades: grade 0 (normal), grade 1 (spinal hyperesthesia with normal gait), grade 2 (ambulatory para/tetraparesis or ataxia), grade 3 (nonambulatory para/tetraparesis), grade 4 (para/tetraplegia with intact nociception), and grade 5 (para/tetraplegia with loss of deep nociception) [17].

### 2.3. Experimental Design

**Figure 3:**
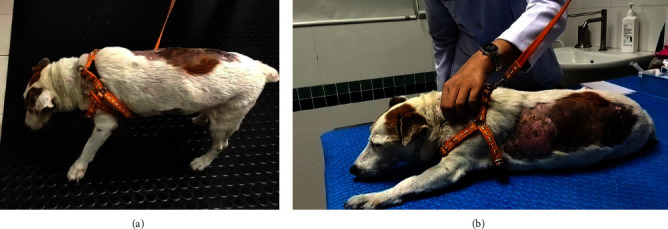
Evaluation of neurological signs: (a) dogs with cervical intervertebral disc disease suffer from severe cervical hyperesthesia showing head pressing posture; (b) examination to detect cervical hyperesthesia.

The dogs suffering from cervical intervertebral disc disease with the clinical sign of cervical hyperesthesia (Figure 3(a)) were divided into two groups of five dogs. G1 was orally treated with 4 mg/kg pregabalin (T. O. Chemical, Ltd., Thailand) twice daily [18], while G2 was treated with 15 mg/kg *Macleaya* extract (leaf, flower, and trunk extract of *Macleaya cordata*; Med Daylight Co., Ltd., Thailand) orally once daily after [19]. Both groups were treated with their specific drugs for 14 days. Then, the ST levels were evaluated at days 0, 7, and 14 posttreatment using the filament VFA technique. The neurological signs, including cervical hyperesthesia and grade of paresis, were assessed at each time point (Figure 3(b)). Blood samples (*n* = 20) were collected to evaluate the changes in hematology and blood chemistry profiles such as ALT, ALP, SCR, BUN, and TP pre- and posttreatment on days 0 and 14.

### 2.4. Statistical Analysis

Sensory thresholds evaluated using filament VFA, hematological, and blood chemistry values at different time points were analyzed based on one-way repeated measures ANOVA, and comparisons were made between the two groups. A *p* value of less than 0.05 was considered statistically significant, and data were presented as the mean ± SEM. Statistical analysis was performed using the NCSS 2021 version 21.0.4 software (NCSS, LLC, USA) [20].

## 3. Results

### 3.1. Dog Characteristics

This study recruited 10 dogs with spinal hyperesthesia. The dog characteristics consisted of six mixed breeds, 1 Bull Terrier, 1 Labrador Retriever, 1 Chihuahua, and 1 Terrier. The male-to-female ratio was 5 : 5, with an age ranging from 4 to 18 years (11.5 years). The body weights ranged from 5.5 to 28 kg (14.5 kg).

### 3.2. Neuropathic Sensory Threshold Evaluation

The VFA technique for evaluating ST was applied to the left forelimb (LF), right forelimb (RF), left hindlimb (LH), and right hindlimb (RH) separately, and the mean was calculated for the 4 limbs in both groups on days 0, 7, and 14 postadministration of the pregabalin or the *Macleaya* extract. The results showed that there were no significant differences in ST values for both groups on days 0, 7, and 14 (Table 1). In G1, the ST values for LF, RF, and RH were not significantly different, while the ST values for LH (*p* = 0.005) and the mean ST value based on all limbs (*p* = 0.017) in the periods were significantly different. In G2, the ST values of RF and RH were not significantly different on days 0, 7, and 14, whereas the ST values of LF (*p* = 0.028), LH (*p* = 0.036), and mean for all limbs (*p* = 0.002) were significantly different at those time points. In addition, the grades used to assess the severity of neurological problems of the two groups showed improved paretic grades at each time point (Table 2).

### 3.3. Hematological and Blood Chemistry Evaluation

There were no significant differences in the hematological and blood chemistry evaluations between days 0 and 14 in both groups (Table 3). The mean SCR level tended to increase posttreatment with pregabalin, whereas G2 did not show this phenomenon. Notably, the levels of ALT and SCR tended to decrease posttreatment with *Macleaya* extract.

## 4. Discussion

We assessed neuropathic pain in dogs evaluated based on filament VFA via ST levels. The detection of neuropathic pain was performed using a neurological examination. Neuropathic pain is characterized by allodynia and hyperesthesia that results in a reduction in ST caused by a disorder of the sensory nervous system [21]. Our study demonstrated that treatment using *Macleaya* extract compared to pregabalin for neuropathic pain relief in dogs could reduce neuropathic pain based on a decrease in the ST value in both groups on days 7 and 14 compared to day 0. Pregabalin and *Macleaya* extract oral administration showed similar efficacy in neuropathic pain reduction. However, the *Macleaya* extract treatment showed a rapid onset and reduced the ST level at day 7, while pregabalin showed a therapeutic effect on day 14, similar to another study involving pregabalin treatment of neuropathic pain in dogs with syringomyelia [22]. This finding demonstrated that *Macleaya* extract could reduce neuropathic pain in the acute phase and may be used in cases of chronic pain as well. In both groups, there was an improvement in the grade of paresis within 7 days postadministration. Neuropathic pain was initially diagnosed as a neurological disease, particularly in the spinal cord regions, including the cervical, thoracic, and sacral parts of the spinal cord [18]. In this study, the metacarpal and metatarsal areas were tested for evaluating ST levels using filament VFA. Among a variety of neuropathic pain measurements, VFA is the gold standard for evaluating mechanical thresholds [23]. Previous studies demonstrated that VFA was practical and noninvasive in detecting neuropathic pain in dogs [3].

Long-term feeding of a diet containing *Macleaya* extract in healthy dogs was well tolerated and did not have adverse effects [19]. This study evaluated CBC, ALP, ALT, SCR, BUN, and TP posttreatment with pregabalin or *Macleaya* extract on day 14 compared to day 0. We demonstrated that there were no significant differences in the hematological and blood chemistry between pregabalin and *Macleaya* extract on day 14. *Macleaya* extract was safe to give to dogs as shown by the authors in [19]. Notably, there was an increase in the mean SCR values (within the normal level) in the dogs in the pregabalin-treated group. Therefore, renal function should be monitored during long-term treatment with pregabalin as reported in humans using another GABA analog (gabapentin) that caused acute renal failure in a patient with rhabdomyolysis [24]. For clinical applications, safety plays an important role in drug evaluation. It is widely acknowledged that the most detectable adverse effects of pregabalin include sedation and ataxia [25]. These adverse effects were not found in the *Macleaya* extract group which seemed to be safe for the quality of life of dogs with neuropathic pain. We demonstrated that *Macleaya* extract has an equivalent effect to pregabalin in reducing neuropathic pain and has a less adverse effect in dogs with cervical hyperesthesia. Confirmatory trials will be required to investigate the full efficacy of the *Macleaya* extract.

Limitations of this study were that the sample size was too small, and most of the dogs in both groups were not given advanced imaging, particularly magnetic resonance imaging, to determine the cause of spinal hyperesthesia, due to the financial cost limiting the consent of some owners. In addition, long-term clinical evaluation of the effectiveness of the *Macleaya* extract could lead to valuable clinical information about this potentially useful new drug for neuropathic pain treatment. Moreover, the frequency of the *Macleaya* extract administration in this study was once daily. At present, no study has comprehensively assessed the effects of different *Macleaya* extract doses. Accordingly, further research should be conducted to test the robustness of our findings.

## 5. Conclusion

*Macleaya* extract may be a promising rapid-onset drug in the treatment of neuropathic pain due to spinal hyperesthesia with similar efficacy to pregabalin, as well as being safe for liver and kidney functions.

## Figures and Tables

**Table 1:** Comparison of sensory threshold (ST) values between pregabalin and *Macleaya* treatment groups.

Area of measurement	Treatments	Day 0 ST value (mean ± SEM)	Day 7 ST value (mean ± SEM)	Day 14 ST value (mean ± SEM)	*p* value
Left forelimb	G1	188 ± 49.55	260 ± 39.93	276 ± 23.96	0.271
	G2	172 ± 36.60	260 ± 39.93	300 ± 0.00	0.028

Right forelimb	G1	188 ± 31.94	236 ± 41.11	276 ± 23.96	0.186
	G2	236 ± 41.11	276 ± 23.96	300 ± 0.00	0.179

Left hindlimb	G1	148 ± 42.63	276 ± 23.96	300 ± 0.00	0.005
	G2	212 ± 38.71	300 ± 0.00	300 ± 0.00	0.037

Right hindlimb	G1	204 ± 44.82	260 ± 39.93	300 ± 0.00	0.095
	G2	252 ± 29.34	300 ± 0.00	300 ± 0.00	0.129

All limbs	G1	182 ± 26.49	258 ± 27.77	288 ± 11.98	0.017
	G2	218 ± 14.26	284 ± 15.97	300 ± 0.00	0.002

G1; pregabalin-treated group at a rate of 4 mg/kg twice daily, G2; *Macleaya*-treated group at a rate of 15 mg/kg once daily for 14 days.

**Table 2:** Improvement of neurological signs in response to pregabalin and *Macleaya* extract.

Treatment group	Dog no	Modified Frankel score		
		Day 0	Day 7	Day 14
Pregabalin	1	1	1	0
	2	1	0	0
	3	1	0	0
	4	1	0	0
	5	1	0	0

*Macleaya* extract	6	1	0	0
	7	1	0	0
	8	1	0	0
	9	1	0	0
	10	1	0	0

Modified Frankel scores: 0 = normal, 1 = spinal hyperesthesia with normal gait, 2 = ambulatory para/tetraparesis or ataxia, 3 = nonambulatory para/tetraparesis, 4 = para/tetraplegia with intact nociception, and 5 = para/tetraplegia with loss of deep nociception.

**Table 3:** Hematological and blood chemistry profiles at pre- and posttreatment.

Parameter	Pregabalin (mean ± SEM)		*p* value	*Macleaya* extract (mean ± SEM)		*p* value	Normal range
	Day 0	Day 14		Day 0	Day 14		
WBC	9.46 ± 1.56	9.28 ± 1.36	0.859	9.24 ± 1.97	9.78 ± 1.85	0.687	6–17 × 10^3^/mm^3^
HCT	38.56 ± 2.69	38.1 ± 2.97	0.800	48.8 ± 7.37	47.72 ± 8.12	0.081	35–55%
PLT	190.80 ± 510.94	174.20 ± 557.62	0.634	332.20 ± 1,722.62	346.20 ± 1,531.52	0.268	200–500 × 10^3^/*µ*l
BUN	29.2 ± 15.16	22.2 ± 14.61	0.505	25.6 ± 2.41	23.4 ± 2.86	0.223	10–26 mg%
SCR	1.19 ± 0.09	1.33 ± 0.04	0.068	1.13 ± 0.15	1.08 ± 0.11	0.642	0.5–1.3 mg%
ALT	153.6 ± 34.49	120 ± 35.49	0.198	58.4 ± 16.02	40.6 ± 15.42	0.153	6–70 IU/L (37°C)
ALP	687.6 ± 340.15	441.4 ± 362.34	0.316	248.4 ± 122.66	181.2 ± 131.43	0.435	8–76 IU/L (37°C)
TP	6.26 ± 0.60	6.28 ± 0.52	0.960	7.08 ± 0.20	6.48 ± 0.64	0.127	5.3–7.8 gm%

WBC: white blood cell count; HCT: hematocrit; PLT: platelet; BUN: blood urea nitrogen; SCR: serum creatinine; ALT: alanine aminotransferase; ALP: alkaline phosphatase; TP: total protein.

## Data Availability

The data used to support the findings of this study are available from the corresponding author on request.
